# Supplementary material for: The Long Noncoding RNA Transcriptome of Dictyostelium discoideum Development
Source: G3 (Bethesda). 2016 Dec 6;7(2):387–98. doi: 10.1534/g3.116.037150 (PMC5295588; doi:10.1534/g3.116.037150)

**Supplemental Figure 1. Sequencing depth of rRNA and chromosomally mapped reads was comparable between rRNA depletion methods.** (a) The total number of mappable reads (y-axis) is tallied for each library preparation method (x-axis). PolyA refers to previously published mRNA-enriched libraries sequenced with 50bp single-end Illumina chemistry (Rosengarten 2015). Nugen and RiboZero refer to different rRNA depletion methods employed in the current study, which were sequenced with 100 bp paired-end Illumina chemistry. Blue shading indicates reads mapped to chromosomes 1 to 6, whereas red shading demarks reads mapped to the extrachromosomal ribosomal palindrome. (b) The percent of the total reads mapped to ribosomal sequences is shown for each experiment. Error bars represent standard error of the mean of each barcoded library sample (PolyA n = 38, Nugen n = 24, RiboZero n = 6).

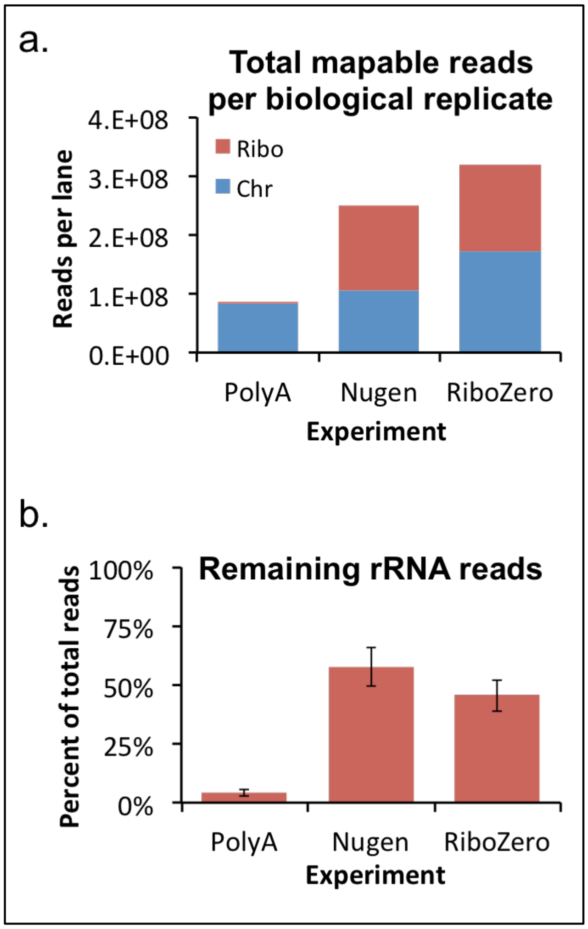

Supplement: Supplementary file 1 [file 387FigureS1.pdf]
