# Supplementary material for: The Long Noncoding RNA Transcriptome of Dictyostelium discoideum Development
Source: G3 (Bethesda). 2016 Dec 6;7(2):387–98. doi: 10.1534/g3.116.037150 (PMC5295588; doi:10.1534/g3.116.037150)
Supplement: Supplementary file 2 [file 387FigureS2.pdf]

Supplemental Figure 2.

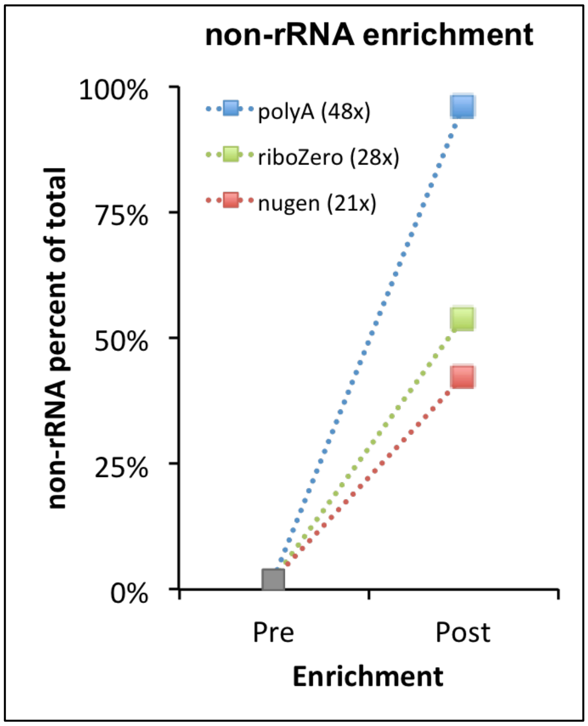

Ribosomal RNA depletion leads to > 20-fold enrichment of non-rRNA sequencing coverage. The fraction (%) of non-rRNA compared to total RNA (y-axis) is plotted as a function of enrichment (x-axis). Enrichment of non-rRNA transcripts was calculated as the fold-increase from 2% pre-treatment to Y% post-treatment, assuming that rRNA constitutes 98% of the total cellular RNA. The different experimental treatments are indicated in the legend, with fold enrichment in parentheses.
