# Supplementary material for: The Long Noncoding RNA Transcriptome of Dictyostelium discoideum Development
Source: G3 (Bethesda). 2016 Dec 6;7(2):387–98. doi: 10.1534/g3.116.037150 (PMC5295588; doi:10.1534/g3.116.037150)
Supplement: Supplementary file 3 [file 387FigureS3.pdf]

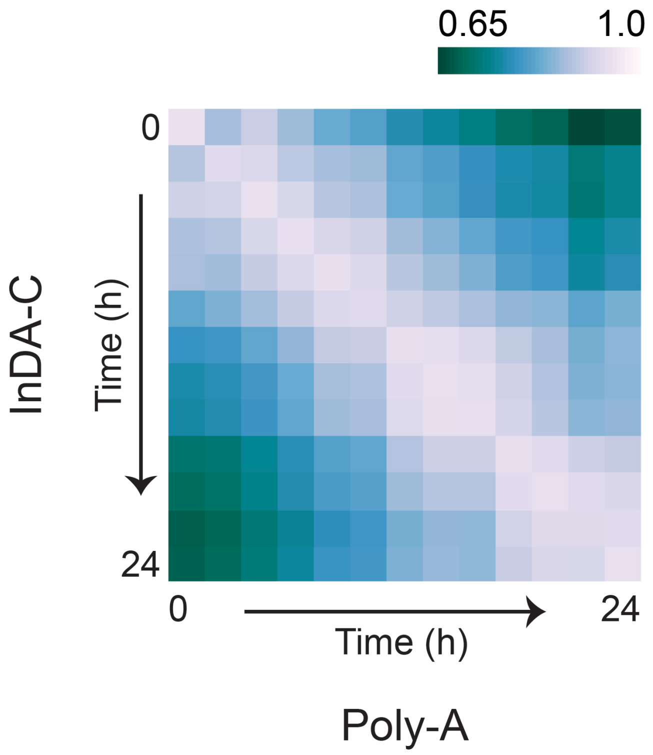

Supplemental Figure 3. Correlation of protein-coding abundances.

mRNA profiles were highly correlated between the same samples prepared by InDA-C rRNA depletion and polyA mRNA enrichment. The y-axis represents data generated in this study from InDA-C libraries, and the x-axis data from poly-A enriched libraries sequenced by Rosengarten et al., 2015. Time points, in hours (h), descend from 0 to 24 on the y-axis, and ascend from left to right on the x-axis. Spearman's correlation values are shown as a heatmap relating mRNA transcriptome profiles at each time point (average of 2 biological replicates). Green shading shows low correlation whereas pink shows high correlation, as indicated in the legend.
