# Supplementary material for: The Long Noncoding RNA Transcriptome of Dictyostelium discoideum Development
Source: G3 (Bethesda). 2016 Dec 6;7(2):387–98. doi: 10.1534/g3.116.037150 (PMC5295588; doi:10.1534/g3.116.037150)
Supplement: Supplementary file 4 [file 387FigureS4.pdf]

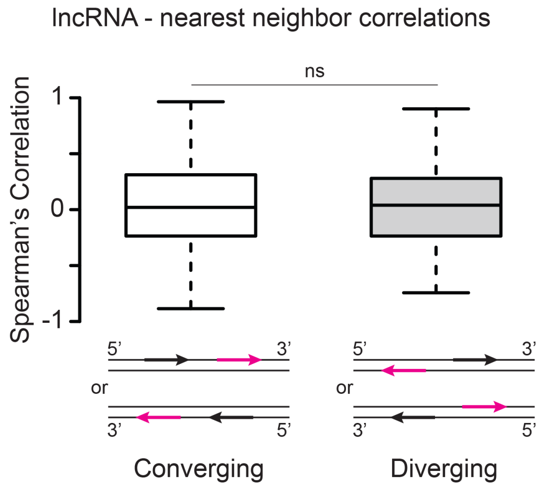

Supplemental Figure 4. Correlation with neighboring gene abundance.

The abundances of most lncRNAs were uncorrelated with the neighboring genes**.** We determined the Spearman's correlation (y-axis) between the temporal transcription profile for each lncRNA and its nearest 5' gene neighbor in either direction (on either strand). The lncRNA orientation (pink arrow) is illustrated relative to the nearest 5' neighbor (black arrow). The arrangement on the left represents "converging" head-to-tail transcription, while the arrangement on the right depicts "diverging" head-to-head transcription. Box height represents the 1st to 3rd quartiles and the horizontal line, the median value. Whisker bars mark 1.5-fold the 1st/3rd quartile range.
