# Supplementary material for: The Long Noncoding RNA Transcriptome of Dictyostelium discoideum Development
Source: G3 (Bethesda). 2016 Dec 6;7(2):387–98. doi: 10.1534/g3.116.037150 (PMC5295588; doi:10.1534/g3.116.037150)
Supplement: Supplementary file 5 [file 387FigureS5.pdf]

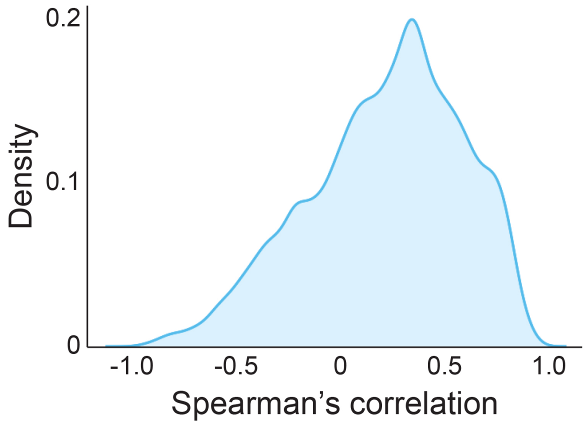

Supplemental Figure 5. Spearman's correlation distribution of asRNA transcript model expression with their sense strand cognates. The median correlation in temporal expression profile between asRNA model and sense-strand mRNA was 0.32. Overall the distribution was shifted positive, although many uncorrelated transcripts were observed as well.
