# Supplementary material for: The Long Noncoding RNA Transcriptome of Dictyostelium discoideum Development
Source: G3 (Bethesda). 2016 Dec 6;7(2):387–98. doi: 10.1534/g3.116.037150 (PMC5295588; doi:10.1534/g3.116.037150)
Supplement: Supplementary file 6 [file 387FigureS6.pdf]

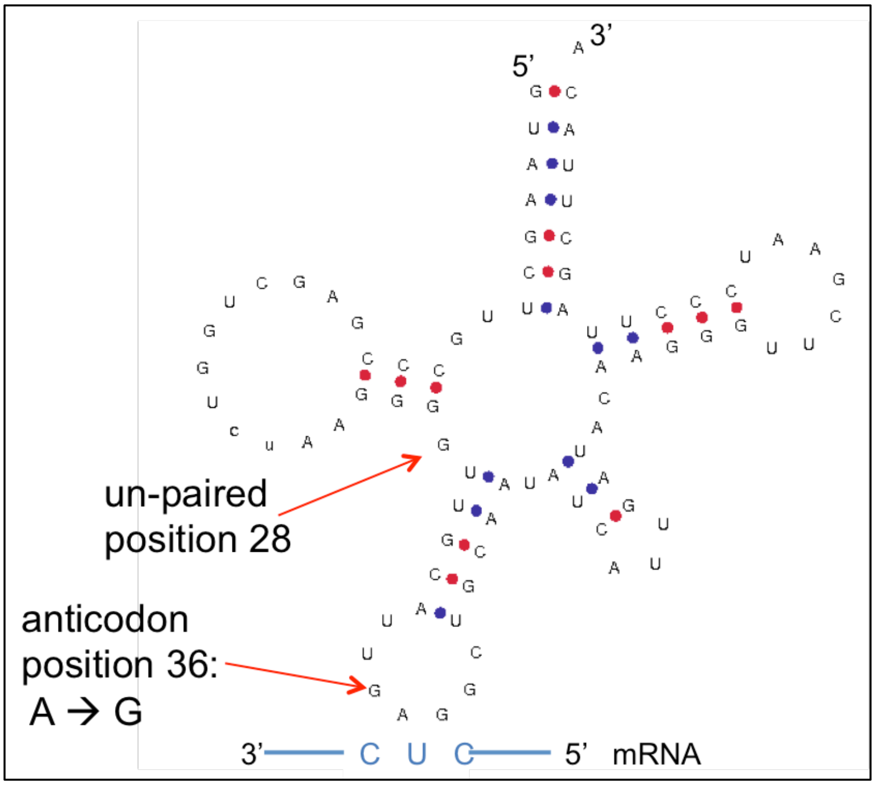

Supplemental Figure 6. tRNA anticodon polymorphism. Predicted tRNA structure of Leucine (CUC) variants illustrates positions of polymorphic bases (red arrows). The variable anticodon base was found at position 36, whereas position 28 does not form a complementary base pair.
