# Supplementary material for: The Long Noncoding RNA Transcriptome of Dictyostelium discoideum Development
Source: G3 (Bethesda). 2016 Dec 6;7(2):387–98. doi: 10.1534/g3.116.037150 (PMC5295588; doi:10.1534/g3.116.037150)
Supplement: Supplementary file 7 [file 387FigureS7.pdf]

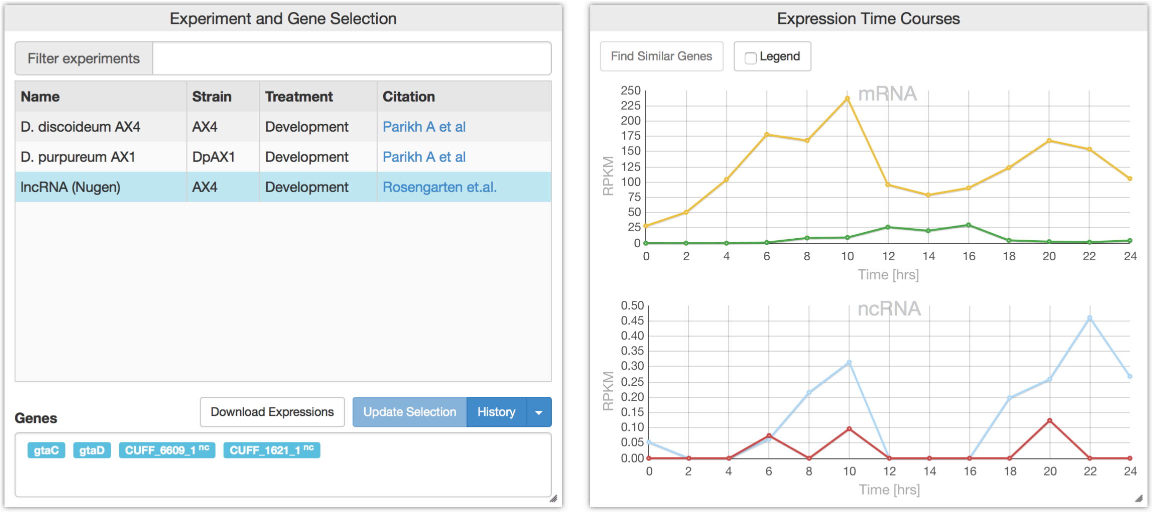

Supplemental Figure 7. ***dictyExpress*** **Time Course Expressions module displays mRNAs and lncRNAs.** Screen shot from the new dictyExpress showing the mRNA – ncRNA split screen time course module. On the left is the Experiment and Gene Selection module, with mRNAs *gtaC* and *gtaD* selected, as well as two lncRNAs identified as having similar time-course profiles. On the right is the Expression Time Courses module with new split-screen functionality to simultaneously plot mRNAs (top) and ncRNAs (bottom).
