# Supplementary material for: The Long Noncoding RNA Transcriptome of Dictyostelium discoideum Development
Source: G3 (Bethesda). 2016 Dec 6;7(2):387–98. doi: 10.1534/g3.116.037150 (PMC5295588; doi:10.1534/g3.116.037150)
Supplement: Supplementary file 8 [file 387FileS1.docx]

File S1. Custom designed InDA-C oligos for rRNA depletion. (.xlsx, 13 KB)

<http://www.g3journal.org/lookup/suppl/doi:10.1534/g3.116.037150/-/DC1/FileS1.xlsx>
