# Supplementary material for: The Long Noncoding RNA Transcriptome of Dictyostelium discoideum Development
Source: G3 (Bethesda). 2016 Dec 6;7(2):387–98. doi: 10.1534/g3.116.037150 (PMC5295588; doi:10.1534/g3.116.037150)
Supplement: Supplementary file 20 [file 387FileS7.docx]

File S7. Spearman’s correlations, strand specificity and intron overlap of asRNA models and sense strand genes. (.xlsx, 1.16 MB)

[http://www.g3journal.org/lookup/suppl/doi:10.1534/g3.116.037150/-/DC1/FileS7.xlsx](http://www.g3journal.org/lookup/suppl/doi:10.1534/g3.116.037150/-/DC1/FileS6.xlsx)
