# Supplementary material for: The Long Noncoding RNA Transcriptome of Dictyostelium discoideum Development
Source: G3 (Bethesda). 2016 Dec 6;7(2):387–98. doi: 10.1534/g3.116.037150 (PMC5295588; doi:10.1534/g3.116.037150)
Supplement: Supplementary file 24 [file 387FileS9.docx]

File S9. Varscan output including all detected tRNA polymorphisms. (.xls, 54 KB)

<http://www.g3journal.org/lookup/suppl/doi:10.1534/g3.116.037150/-/DC1/FileS8.xls>
