# Supplementary material for: The Long Noncoding RNA Transcriptome of Dictyostelium discoideum Development
Source: G3 (Bethesda). 2016 Dec 6;7(2):387–98. doi: 10.1534/g3.116.037150 (PMC5295588; doi:10.1534/g3.116.037150)
Supplement: Supplementary file 26 [file 387ncRNA_supplemental_figures_G3.pdf]

Supplemental Figure 1. Sequencing depth of rRNA and chromosomally mapped reads was comparable between rRNA depletion methods. (a) The total number of mappable reads (y-axis) is tallied for each library preparation method (x-axis). PolyA refers to previously published mRNA-enriched libraries sequenced with 50bp single-end Illumina chemistry (Rosengarten 2015). Nugen and RiboZero refer to different rRNA depletion methods employed in the current study, which were sequenced with 100 bp paired-end Illumina chemistry. Blue shading indicates reads mapped to chromosomes 1 to 6, whereas red shading demarks reads mapped to the extrachromosomal ribosomal palindrome. (b) The percent of the total reads mapped to ribosomal sequences is shown for each experiment. Error bars represent standard error of the mean of each barcoded library sample (PolyA n = 38, Nugen n = 24, RiboZero n = 6).

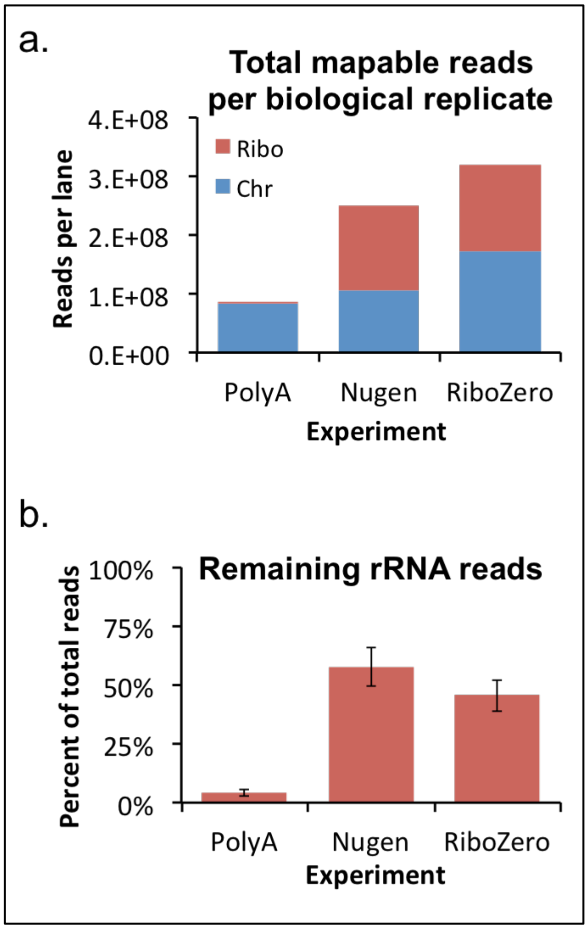

Supplemental Figure 2.

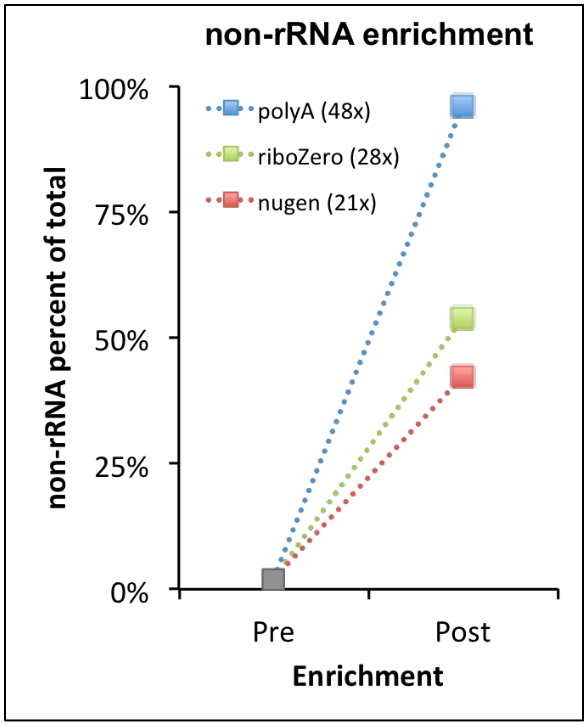

Ribosomal RNA depletion leads to > 20-fold enrichment of non-rRNA sequencing coverage. The fraction (%) of non-rRNA compared to total RNA (y-axis) is plotted as a function of enrichment (x-axis). Enrichment of non-rRNA transcripts was calculated as the fold-increase from 2% pre-treatment to Y% post-treatment, assuming that rRNA constitutes 98% of the total cellular RNA. The different experimental treatments are indicated in the legend, with fold enrichment in parentheses.

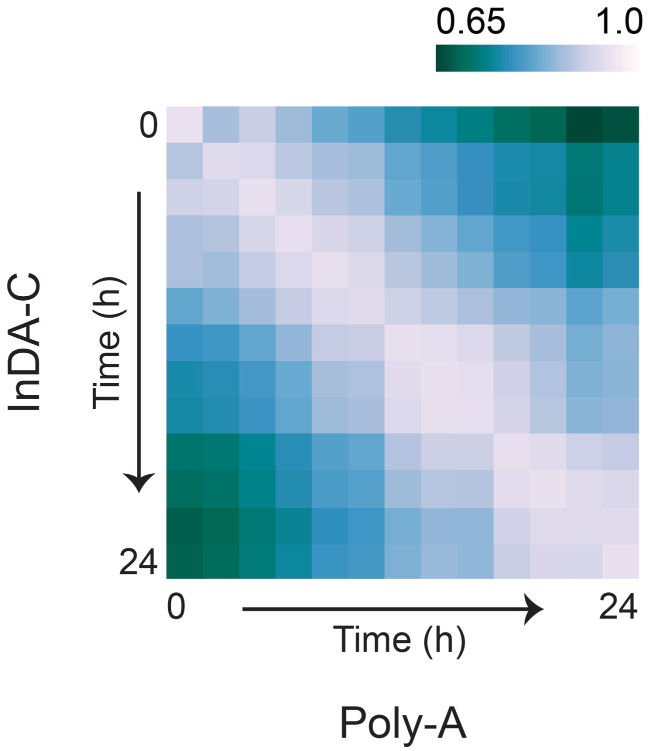

Supplemental Figure 3. Correlation of protein-coding abundances.

mRNA profiles were highly correlated between the same samples prepared by InDA-C rRNA depletion and polyA mRNA enrichment. The y-axis represents data generated in this study from InDA-C libraries, and the x-axis data from poly-A enriched libraries sequenced by Rosengarten et al., 2015. Time points, in hours (h), descend from 0 to 24 on the y-axis, and ascend from left to right on the x-axis. Spearman's correlation values are shown as a heatmap relating mRNA transcriptome profiles at each time point (average of 2 biological replicates). Green shading shows low correlation whereas pink shows high correlation, as indicated in the legend.

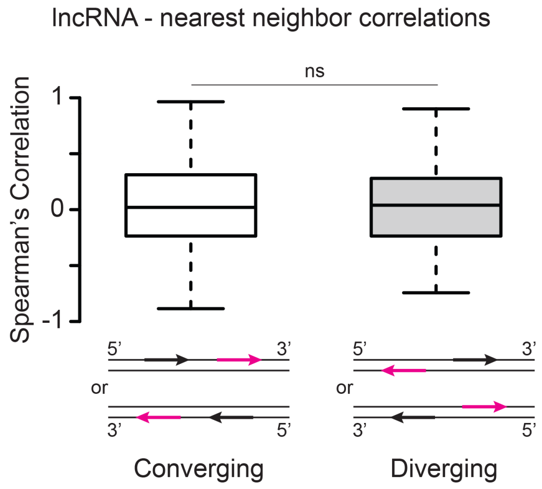

Supplemental Figure 4. Correlation with neighboring gene abundance.

The abundances of most lncRNAs were uncorrelated with the neighboring genes**.** We determined the Spearman's correlation (y-axis) between the temporal transcription profile for each lncRNA and its nearest 5' gene neighbor in either direction (on either strand). The lncRNA orientation (pink arrow) is illustrated relative to the nearest 5' neighbor (black arrow). The arrangement on the left represents "converging" head-to-tail transcription, while the arrangement on the right depicts "diverging" head-to-head transcription. Box height represents the 1st to 3rd quartiles and the horizontal line, the median value. Whisker bars mark 1.5-fold the 1st/3rd quartile range.

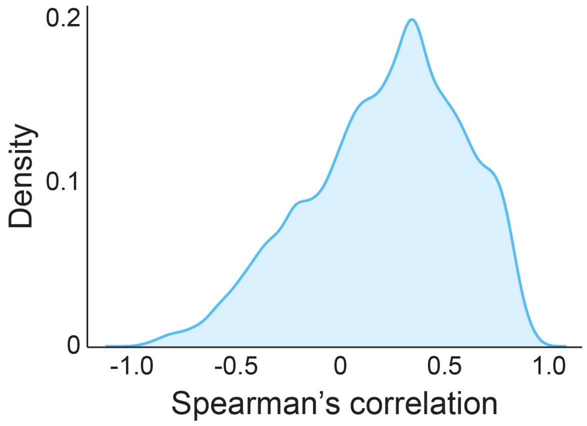

Supplemental Figure 5. Spearman's correlation distribution of asRNA transcript model expression with their sense strand cognates. The median correlation in temporal expression profile between asRNA model and sense-strand mRNA was 0.32. Overall the distribution was shifted positive, although many uncorrelated transcripts were observed as well.

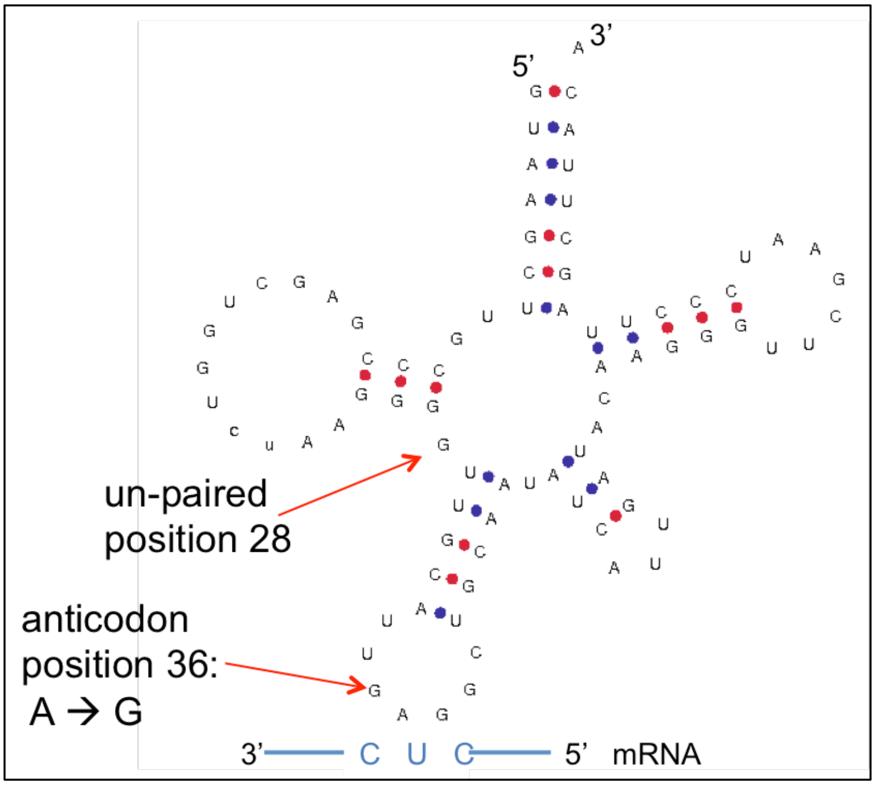

Supplemental Figure 6. tRNA anticodon polymorphism. Predicted tRNA structure of Leucine (CUC) variants illustrates positions of polymorphic bases (red arrows). The variable anticodon base was found at position 36, whereas position 28 does not form a complementary base pair.

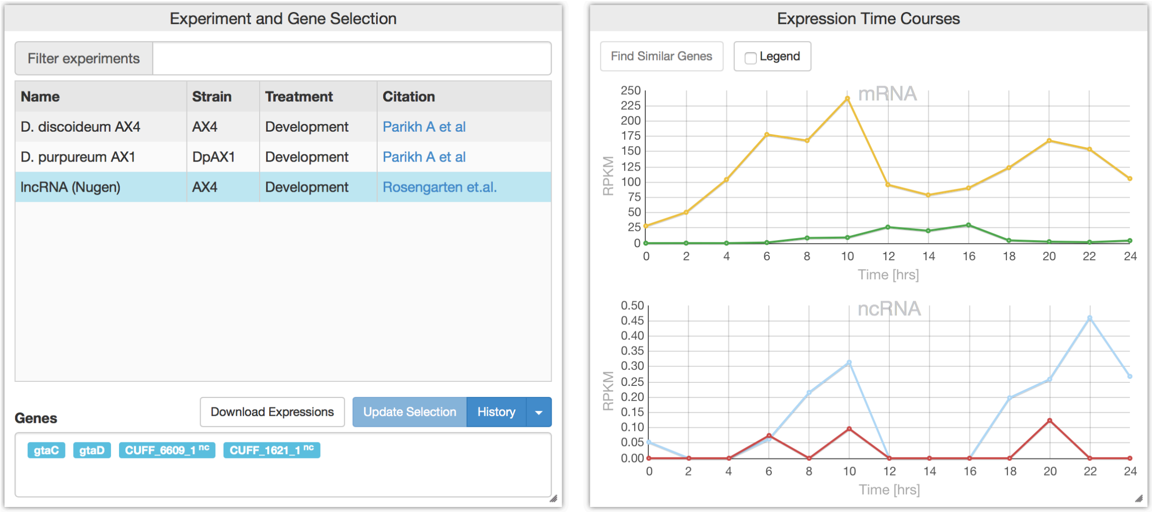

Supplemental Figure 7. *dictyExpress* Time Course Expressions module displays mRNAs and lncRNAs. Screen shot from the new dictyExpress showing the mRNA – ncRNA split screen time course module. On the left is the Experiment and Gene Selection module, with mRNAs *gtaC* and *gtaD* selected, as well as two lncRNAs identified as having similar time-course profiles. On the right is the Expression Time Courses module with new split-screen functionality to simultaneously plot mRNAs (top) and ncRNAs (bottom).
