# Supplementary material for: The Long Noncoding RNA Transcriptome of Dictyostelium discoideum Development
Source: G3 (Bethesda). 2016 Dec 6;7(2):387–98. doi: 10.1534/g3.116.037150 (PMC5295588; doi:10.1534/g3.116.037150)
Supplement: Supplementary file 27 [file 387Rosengarten_Resubmission_20161202.docx]

# Full title

The long non-coding RNA transcriptome of *Dictyostelium discoideum* development

# Short title

*Dictyostelium discoideum* long non-coding RNAs

# Authors

*§^[[1]](#footnote-1)^ Rafael D. Rosengarten (Corresponding author)

email: rafael@genialis.com

*§ Balaji Santhanam

† Janez Kokosar

* Gad Shaulsky (Co-corresponding author)

email: gadi@bcm.edu

* Department of Molecular and Human Genetics, Baylor College of Medicine, Houston, TX 77030, USA

† Computational and Integrative Biomedical Research Center, Baylor College of Medicine, Houston, TX 77030, USA

§ These authors contributed equally.

GEO accession: GSE90829

# Abstract

*Dictyostelium discoideum* live in the soil as single cells, engulfing bacteria and growing vegetatively. Upon starvation, tens of thousands of amoebae enter a developmental program that includes aggregation, multicellular differentiation, and sporulation. Major shifts across the protein-coding transcriptome accompany these developmental changes. However, no study has presented a global survey of long non-coding RNAs in *D. discoideum*. To characterize the antisense and long intergenic non-coding RNA transcriptome, we analyzed previously published developmental time course samples using an RNA-sequencing library preparation method that selectively depletes ribosomal RNAs. We detected the accumulation of transcripts for 9,833 protein-coding messenger RNAs, 621 long intergenic non-coding RNAs and 162 putative antisense RNAs. The non-coding RNAs were interspersed throughout the genome, and were distinct in expression level, length and nucleotide composition. The non-coding transcriptome displayed a temporal profile similar to the coding transcriptome, with stages of gradual change interspersed with larger leaps. The transcription profiles of some non-coding RNAs were strongly correlated with known differentially expressed coding RNAs, hinting at a functional role for these molecules during development. Examining the mitochondrial transcriptome, we modeled two novel antisense transcripts. We applied yet another ribosomal depletion method to a subset of the samples to better retain tRNA transcripts. We observed polymorphisms in tRNA anticodons that suggested a post-transcriptional means by which *D. discoideum* compensates for codons missing in the genomic complement of tRNAs. We concluded that the prevalence and characteristics of long non-coding RNAs indicate these molecules are relevant to the progression of molecular and cellular phenotypes during development.

**Key words:** transcriptome time course, development, non-coding RNA, ribosomal RNA depletion, *Dictyostelium discoideum*, slime mold

# Introduction

The social amoeba *D. discoideum* has captured the imagination of biologists for over 75 years (Raper 1940; Williams 2010). Much of the fascination is due to its unusual life cycle, which is divided between a single-celled, vegetative growth stage and a tightly regulated multicellular developmental program. Development is triggered by starvation, and proceeds through various stages: chemotaxis and aggregation, multicellular differentiation, morphogenesis and reproductive maturation (reviewed in (Kessin 2001)). Many cellular and molecular events critical to development have been elucidated through genetic and chemical experiments. The growing adoption of high throughput sequencing adds a powerful complementary approach to analyzing the regulation of this process (Loomis and Shaulsky 2011).

The developmentally regulated, protein coding transcriptome of *D. discoideum* has been characterized extensively (Van Driessche *et al.* 2002; Iranfar *et al.* 2003; Parikh *et al.* 2010; Rosengarten, *et al.* 2015). Roughly two thirds of the gene models present in the genome are expressed to some extent during development. Critical transcription regulatory networks are coming into relief with the combination of genetic screens and deep sequencing data (Cai *et al.* 2014; Santhanam *et al.* 2015). In many model organisms, non-coding (nc)RNAs such as long intergenic ncRNAs (lncRNAs) and antisense (as)RNAs also play important roles in the regulation of gene expression (Qu and Adelson 2012; Guil and Esteller 2012; Pelechano and Steinmetz 2013).

The presence of various types of ncRNAs in *D. discoideum*, beginning with nuclear RNAs, has been appreciated for decades (Takeishi and Kaneda 1979, 1981). One of the first eukaryotic examples of endogenous antisense regulation of an mRNA cognate (the prespore gene *psvA*) was demonstrated in *D. discoideum* (Hildebrandt and Nellen 1992)*.* Over the years, construction of small insert cDNA libraries, de novo computational searches, and various deep-sequencing approaches have identified novel classes of developmentally important small ncRNAs and microRNAs (Aspegren *et al.* 2004; Hinas *et al.* 2006; Larsson *et al.* 2008; Avesson *et al.* 2011, 2012). Nevertheless, we have yet to thoroughly catalog the identities of lncRNAs and asRNAs, and to examine how their abundances relate to developmental changes.

Herein we describe the first comprehensive annotation of lncRNA transcript models, and identify a set of putative asRNAs, in the *D. discoideum* genome. These transcripts were identified by bioinformatics analysis of RNA-sequencing (RNA-seq) data from ribosomal RNA (rRNA) depleted libraries. We used the same biological samples as in an earlier published developmental time course (Rosengarten *et al.* 2015a). However, the current rRNA depletion strategy deviates from the previous poly-A selection in that it should retain non-poly-adenylated transcripts in addition to mRNAs. Our analyses identified hundreds of intergenic lncRNA loci. While these were typically expressed at much lower levels than mRNAs, their abundance followed similar temporal patterns over the course of development. Strong correlation was observed between the temporal pattern of a few dozen lncRNAs and mRNAs abundant in both early and late development. We further examined other non-poly-adenylated RNAs, including the first deep sequencing analysis of the mitochondrial transcriptome. Analysis of tRNA expression provided evidence for post-transcriptional modifications that compensate for various anti-codons missing from the genomic tRNA complement. The widespread expression of lncRNAs during *D. discoideum* development suggests that future genetic studies should consider the effects of intergenic elements more closely.

# Materials and Methods

## Growth, development and sample collection

In this study, we processed aliquots of total RNA that had been collected in Rosengarten et al. (2015a). Briefly: *D. discoideum* cells (strain AX4) were grown in HL-5 nutrient medium, shaking at 22° to mid-log phase. Cells were developed on nitrocellulose filters (5x10 cells per 5 cm filter) saturated in PDF buffer for 24 hours, as described in (Miranda, Zhuchenko, *et al.* 2013). Every one to two hours, developing cells were scraped into 1 mL Trizol reagent (Life Sciences). Total RNA was isolated by phenol chloroform extraction and ethanol precipitation. Two biological replicates were collected for each time course. The previous analysis found little difference in population-average gene expression at 1-hour versus 2-hour time resolution (Rosengarten, *et al.* 2015). Therefore we selected samples 2-hours apart, from 0h to 24h.

## cDNA library preparation and RNA-sequencing

We constructed multiplexed RNA-sequencing libraries using the Ovation Universal RNA-Seq System (Nugen, Carlsbad, CA) to exclude ribosomal RNA and enrich other RNA species according to the manufacturer’s recommended protocol. For each sample, 200 ng total RNA was annealed to random and oligo-dT primers and treated with heat-labile arctic double stranded DNase, prior to first- then second-strand cDNA synthesis. The resulting cDNA was fragmented by sonication using the Covaris S-series System with the recommended settings to achieve 150 - 200 bp median fragments. cDNA was recovered using magnetic beads with 2 ethanol wash steps, followed by enzymatic end repair of the fragments. Next, barcoded adapters were ligated to each sample, followed by an enzymatic strand selection step and magnetic bead recovery, as above. rRNAs were targeted for depletion by the addition of custom designed oligonucleotides specific for the 28s, 17s, 5.8s and 5s rRNA genes, as well as the mitochondrial large and small RNA subunits (rnlA and rnsA, respectively). A list of these depletion probes is provided in supplemental file 1. The next step was Insert Dependent Adaptor Cleavage (InDA-C) to remove the adapters containing priming sites from all targeted rRNA molecules. RT-PCR was used to determine that 15 cycles were required for the subsequent library amplification.

To examine tRNA abundance, we used the Ribo-Zero Plant Seed/Root magnetic kit (Epicentre), designed to retain molecules smaller than 100 bp. For this we chose six samples from biological replicate 1 (0, 4, 8, 12, 16 and 22h). Library preparation was performed according to manufacturer’s recommended protocol.

The full time course InDA-C and the sub-sample Ribo-Zero cDNA libraries were sequenced by Illumina Hiseq2500 with paired ends and read length of 100 base pairs (bp).

## Primary sequence analysis

We checked sequencing data quality using FastQC (v0.10.1) (Andrews 2010). We eliminated three samples—biological replicate 2: 12h, 16h and 22h—that did not meet our quality criteria. For all other samples, sequences were aligned to the *D. discoideum* reference genome (Miranda, Rot, *et al.* 2013) by providing strand information when available using TopHat (v2.0.13) (Trapnell *et al.* 2009). We only permitted uniquely mapped reads and supplied reference transcript annotations (<http://dictybase.org/> version 2013) (*--mate-inner-dist 100 --mate-std-dev 20 --num-threads 4 --GTF -g 1 --report-secondary-alignments --microexon-search --no-mixed --no-discordant --min-intron-length 70 --max-intron-length 500*). We assembled transcripts for each sample using Cufflinks (v2.2.1) (Trapnell *et al.* 2012) with the option *--GTF-guide* to guide the reference annotation based transcript (RABT) assembly. The transcript annotation files from cufflinks were then merged using Cuffmerge (v2.2.1) to obtain a final transcriptome assembly (File S2). By comparing these transcripts to the existing transcript annotations, we identified putative non-coding transcripts. The above primary analyses were all performed using pipelines written for Genialis GenBoard software. We used Transdecoder (v2.0.1) (Haas et al. 2013) with domain homology search options and ORF length thresholds of 25, 50 and 100 amino acids to estimate the coding potential of the final RABT assembled transcriptome.

## Transcriptome analysis

We quantified the transcript abundance for all transcripts contained in the final transcript set by counting uniquely mapped reads and accounting for the strand information where available. We standardized transcript abundance by accounting for the mapable lengths of transcripts and the total number of mapped reads (excluding those mapped to the ribosomal palindrome chrR) in each experiment. Long noncoding and antisense transcript models were filtered according to the following criteria: minimum length of 200 bp; at least two raw reads per transcript model at any time point; mapable expression greater than zero at any time point; and lncRNAs must reside entirely within intergenic regions with no tiling path to a neighboring gene.

Antisense models were further filtered to remove possible artifacts from template strand switching by removing all those transcripts whose tiling path gaps exceeded 5% of the read coverage, typically corresponding to sense-strand introns. Tiling paths were determined for models that were supported by properly paired reads of CIGAR (Compact Idiosyncratic Gapped Alignment Report) 100M. Strand specificity was calculated at 9,833 protein coding loci that remained after filtering for minimum expression values. For each mRNA transcript with a putative antisense transcript, we defined a composite contiguous genomic region that fully included both gene models. On this region we calculated strand specificity as the fraction of total reads that align to the sense strand. Strand specificity was measured on the aggregate of the entire stranded InDA-C data and only included properly paired reads. Spearman’s correlation of the temporal expression profiles between sense and anti-sense transcripts was determined by comparing their standardized average transcript abundances across all time points. Additionally, we also manually inspected read coverage patterns of the aggregated InDA-C data using the Integrated Genomics Viewer (IGV) (Robinson *et al.* 2011; Thorvaldsdóttir *et al.* 2013). These metrics are reported (File S7) to enable researchers to prioritize asRNA models for future validation, though the asRNA transcripts are excluded from the statistical characterizations herein.

A table of curated transcripts and their abundances are provided in File S3. Transcript density was calculated as a proportion of the base pairs per 10 kb stepping window and plotted using Circos (Krzywinski *et al.* 2009). Relative distances between the transcriptomes were visualized using classical multi-dimensional scaling (R function cmdscale) and examined using hierarchical clustering with bootstrapping (R package ‘pvclust’ version 1.2-2 (Suzuki and Shimodaira 2006) with optimized leaf ordering; R package ‘cba’ version 0.2-14). We used Spearman’s correlation (SC) to calculate the distance (D = 1-SC) and complete linkage as the clustering criterion. Heatmaps were generated with the visual programming software suite Orange (Demšar *et al.* 2013).

Rosengarten et al. (2015a) identified 3197 protein-coding genes that were differentially upregulated (FDR < 0.01; >= 2-fold) during development compared to the 0 hr time point. We used transcript abundance from the InDA-C library (Ovation, Nugen) data of these 3197 coding transcripts and 622 long non-coding transcripts to compute pairwise Spearman correlations with adjustment for multiple tests (R package psych, function “corr.test”). Correlation coefficients that met the statistical threshold of FDR < 0.01 were visualized as a heatmap (R package gplots, function heatmap.2).

## tRNA analysis

Most genes encoding tRNAs are present in the *D. discoideum* genome in multiple copies (Eichinger *et al.* 2005). We extracted all the unique sequences of tRNA genes and created a new reference genome with each representing a separate contig. Since tRNAs are typically between 70-95 bp, we trimmed our reads by 65 bp from the 3’ end before mapping. The resulting 35 bp paired-end reads were mapped as single-end reads using bowtie2 (v2.2.3) (Langmead and Salzberg 2012) permitting unique matches. Using samtools (v1.3) (Li *et al.* 2009), we first aggregated the resulting bam files from all 6 time points and then using the “mpileup” function, created a pileup. To identify variants in the tRNA transcriptome, we used Varscan (v2.3.9) (Koboldt *et al.* 2012) and identified 64 putative variants, allowing for p-values < 0.25.

## REMI mutant phenotyping

Mutant *D. discoideum* strains with lesions in or immediately adjacent to putative lncRNA loci were identified from a local database of libraries of barcoded random insert mutants. Fifteen of these strains were recovered from frozen stocks and grown on SM-agar with lawns of *Klebsiella pneumonia* at 22°. These strains were allowed to clear the bacteria and develop. Six of these strains were transferred to nutritive media, grown to mid-log, and developed on nitrocellulose filters, as described above.

## Data availability

Gene expression data are available at GEO with the accession number: GSE90829. File S2 contains the raw transcriptome assembly output by cufflinks/cuffmerge. File S3 contains the transcript abundances of these genes and DDB_G gene models from the latest genome assembly. File S4 contains a table of all curated ncRNA transcripts and their corresponding DDB_G gene model. File S5 contains the mRNA expression values used to determine correlations between library preparation methods. File S6 contains Spearman’s correlations and strand orientation between lncRNAs and their nearest neighbors. File S7 contains asRNA confidence statistics. File S8 contains a table of REMI mutant strains, which are available upon request. File S9 contains the Varscan output from the tRNA analysis. Further, upon publication all transcriptome data from this study may be explored and compared to previous works at [www.dictyexpress.org](http://www.dictyexpress.org).

# Results and Discussion

## Strand-specific ribosomal RNA depleted libraries are consistent with poly-A enriched benchmarks

The protein coding transcriptome of *D. discoideum* has been characterized extensively, most recently by exploring changes in mRNA abundance every one to two hours over the 24-hour course of development (Rosengarten *et al.* 2015a). To characterize the noncoding portion of the transcriptome, we analyzed aliquots of the same two-hour samples, comprising two biological replicates. RNA was prepared for RNA-sequencing by enzymatic depletion of ribosomal RNA (rRNA) using the Insert Dependent Adapter Cleavage (InDA-C) method (Nugen, Carlsbad, California) with custom designed rRNA oligonucleotides. We constructed strand-specific libraries using the Ovation kit (Nugen) and sequenced them by 100 bp paired-end Illumina chemistry. Ribosomal RNA constitutes around 96 - 98% of cellular RNA in *D. discoideum* (Sucgang *et al.* 2003), but after processing only 40 - 60% of the sequencing reads mapped to rRNA genes, confirming an enrichment of non-rRNA in these libraries and facilitating deep-coverage RNA-seq analysis (S1 and S2 Figs).

We next validated the quality of the rRNA-depleted libraries by comparing the mRNA profiles to those previously characterized by poly-A selection (Rosengarten *et al.* 2015a). These experiments utilized identical biological samples, but were processed and sequenced using different technologies. In the poly-A libraries we detected transcript abundance for 10,010 protein-coding genes at some point in development. The same minimal criterion included 9,833 genes from the new libraries, 99% of which overlapped with the previous set (File S5). We calculated Spearman’s correlations of the mRNA abundances at each sample from the two experiments (S3 Fig), and observed a mean correlation of 0.96 for corresponding time points. We conclude that the stranded rRNA depletion libraries are representative of the mRNA transcriptome, in addition to enabling the quantification of various long non-coding RNAs, namely antisense RNA (asRNA) and intergenic long non-coding RNA (lncRNAs).

## Long noncoding RNAs are dispersed throughout the genome

Analyses of the RNA-seq data identified lncRNAs that reside entirely within intergenic regions with no contiguous tiling path to neighboring genes. The transcript models were filtered for minimum length and coverage, as described above. In total we identified 621 lncRNAs with measurable expression at some point during growth or development (Fig 1a, Files S3 and S4). We detected open reading frames 150 bp or longer in only ~10% of the lncRNAs models, indicating most of these transcripts do not have substantial coding potential. Genomic segments encoding lncRNAs were found interspersed among protein-coding loci with no obvious pattern of clustering (Fig 1a). This distribution contrasts with small noncoding RNAs and tRNAs, which are often found in clusters in the *D. discoideum* genome (Aspegren *et al.* 2004; Eichinger *et al.* 2005; Hinas and Söderbom 2007).

Putative asRNAs were defined as those transcripts whose mapping overlapped some portion of a known gene model encoded on the opposite strand, and were filtered for length and coverage as well. We observed considerable correlation in transcript abundance with sense-strand cognates (S5 Fig), as well as troubling similarities in splice patterns that suggested many of the asRNA models were artifacts. We further characterized the strand specificity of the asRNA models, filtered those with tiling path gaps characteristic of sense-strand artifacts. However, due to our inability to consistently distinguish between true antisense transcription and strand-switching products, asRNA models are excluded from the statistical characterizations below. Instead they are included in the supplemental files, available for future validation (Files S3, S4 and S7). One notable exception is discussed below.

Because we impose a filter on the lncRNAs models removing those with tiling paths to the nearest neighbor, we do not believe our transcript models to be artifacts resulting from transcriptional read through. Nevertheless, the lncRNAs might represent transcripts that are expressed from a shared upstream promoter and subsequently processed from the protein-coding transcript. To determine the degree to which lncRNAs show similar expression profiles with their 5’ neighbor, we calculated the Spearman’s correlation for each lncRNA and its nearest neighbors on both sides, and grouped these based on the neighbor’s orientation (S4 Fig, File S6).

Three quarters of all converging lncRNA-neighbors were found to have a Spearman’s correlation less than 0.30, and the median lncRNAs correlation (0.02) was lower than that of 1,000 randomly sampled genes (median = 0.08, Mann Whitney U-test, p = 0.05). Thus, while a subset of putative lncRNAs is co-expressed with their 5’ converging neighbor, most lncRNAs are expressed independently. To test for potential bi-directional promoter activity, we similarly measured the correlations between lncRNA models and their diverging 5’ neighbor on the opposite strand. Here we observed a median correlation of 0.03 for lncRNAs, slightly lower than expected at random (Mann Whitney U-test, p = 0.02). Though some lncRNAs are co-expressed with their divergent neighbor, we reject the hypothesis that bidirectional promoter activity is a general driver of lncRNA expression.

Transcriptional read-through is not the only mechanism that could account for the minority of examples of lncRNA-neighbor pairs with strong correlations in abundance. One hypothesis might be a cis-regulatory effect of the lncRNA on its gene neighbor. Alternatively, co-expression might represent a more transcriptionally active chromatin state (Rinn and Chang 2012; Guil and Esteller 2012; Kornienko et al. 2013; Quinn and Chang 2016).

Noncoding RNAs were considerably less abundant than mRNAs. The median maximum expression at any time point was 41 reads per kilobase per million (RPKM) for mRNAs and 1.0 RPKM for lncRNAs (Fig 1b). The lower abundance of noncoding transcripts might be due to lack of strong promoters, or to effects of poly-adenylation on mRNA stability (Bernstein *et al.* 1989; Sachs 1990; Wang *et al.* 1999). The relative maximum abundance of different classes of RNAs is consistent with that observed in other organisms, such as the malaria vector *Plasmodium* *falciparum*, and even in humans (Derrien *et al.* 2012; Broadbent *et al.* 2015). Derrien and coworkers (2012) cataloged a comprehensive annotation of human lncRNAs for the GENCODE consortium, with median expression differences around 2 orders of magnitude between mRNA and lncRNAs across many different tissue types. Recent experimental evidence suggests that lower lncRNA abundances in bulk-cell sequencing samples is due not to lower levels of expression, but rather greater cell-to-cell variation in expression. Single-cell RNA-seq analysis in the neurocortex revealed that lncRNAs are expressed at levels comparable to mRNAs, but are expressed in a much lower fraction of cells (Liu et al. 2016). Thus bulk analysis results in a lower average abundance. In our study, as in other bulk analyses, each class of lncRNA did include numerous transcripts with maximum abundance levels more akin to mRNAs, although the median for lncRNA was lower (Fig 1b). It will be interesting to see what the first single-cell RNA-seq studies in *Dictyostelium* reveal regarding cellular lncRNA heterogeneity.

lncRNA transcript models were considerably shorter than typical mRNAs (median = 628 vs. 1400 bp) (Fig 1c). The relative shortness of the lncRNAs is not surprising, because these are constrained by the overall available intergenic space, which in *D. discoideum* is only roughly 700 bp on average (Eichinger et al. 2005). Twenty lncRNAs were modeled to have as many as three exons, but splice products remain to be verified.

The *D. discoideum* genome is very AT-rich, but protein coding regions (i.e. ORFs), are more GC rich than intergenic regions (Eichinger *et al.* 2005). The median GC content of ORFs was 25%, whereas lncRNAs GC content was 17% (Fig 1d). The nucleotide composition of non-coding transcripts is similar to that described for *P. falciparum*, which also has a highly AT-skewed genome (Broadbent *et al.* 2015). Intergeneic regions of the *D. discoideum* genome exhibit a strong AT-bias, low complexity and long homo-polymer tracts. We asked whether the lncRNAs were distinct in nucleotide composition from non-expressed intergenic segments. Indeed, randomly selected intergenic sequences were 13% GC on average, significantly lower than lncRNAs (Fig 1d). This difference provides additional confidence in the lncRNA transcript models. One might speculate that some lower limit in GC-content prevents RNA polymerase and associated machinery from transcribing regions below some minimum complexity or GC composition. Anecdotal and published accounts consistently report struggles in amplifying intergenic DNA from *Dictyostelium* (Rosengarten *et al.* 2015b; Eichinger *et al.* 2005). Perhaps these in vitro difficulties reflect challenges experienced by the amoebae themselves.

## Temporal changes in long noncoding RNA abundances follow a similar trajectory as that of the mRNA transcriptome

The protein coding transcriptome of *D. discoideum* changes dramatically over the course of development, with major shifts in the population-average transcript abundances during starvation, multicellular integration and differentiation, and again from the culmination of slugs to fruiting bodies (Rosengarten *et al.* 2015a; Van Driessche *et al.* 2002; Parikh *et al.* 2010). The abundances of mRNA and lncRNA transcripts were examined in heatmaps (Fig 2a,b). In this view, each row represents a transcript, color-coded to show relative changes in abundance for that molecule. Consistent with previous studies (Rosengarten *et al.* 2015b; Van Driessche *et al.* 2002; Parikh *et al.* 2010), the mRNA underwent dramatic changes in expression over the time course (Fig 2a). Likewise, we found that the noncoding transcriptome also changes over developmental time (Fig 2b). Numerous genetics studies have shown that transcriptome dynamics are regulated and have important phenotypic consequences (Williams 2006; Cai *et al.* 2014; Santhanam *et al.* 2015). For example, deletion of the transcription factor encoding *gtaC* manifests itself in an arrest of both the developmental transcriptome state and morphological progression (Cai *et al.* 2014). Thus we hypothesize that the dynamic transcription of long noncoding RNAs may also contribute to development at the (multi-) cellular level.

Multidimensional scaling (MDS) is a powerful approach to visualize high dimensional data in lower dimensional space. MDS can be used to visualize the relative differences between transcriptomes (time points), wherein the Euclidean distances between points in 2-dimensional space correspond to overall dissimilarity between entire transcriptomes of those samples. This analysis revealed the lncRNA temporal changes followed a similar pattern to that of the mRNA, previously described as clusters of slowly changing stages punctuated by gaps representing larger changes in the molecular phenotype (Rosengarten *et al.* 2015a) (Fig 2c,d). Transcriptomes in MDS dimension 1 were nearly collinear with time, although the relationship was imperfect for the lncRNAs (Fig 2e,f).

From the MDS analysis we observed a large temporal shift in this library between 10 and 12 hours of development, and bigger still between 16 and 18 hours (Fig 2d-f). The previous poly-A-based analysis of these samples reported the greatest single transcriptome change between 10 and 12 hours, and also observed considerable separation between the 16 and 18 hour transcriptomes (Rosengarten *et al.* 2015a). Both of these time frames coincide with major morphological changes. The observed signal is robust to library preparation method and is recapitulated by both mRNA and lncRNAs datasets. The consistency in transcriptome pattern among both classes of RNA lends further support to the characterization of the developmental transcriptome as a global quantitative phenotype (Van Driessche *et al.* 2005). Further investigations into the gene regulatory pathways in *D. discoideum* might consider noncoding RNA as well as mRNA responses to genetic perturbations and transcription factor binding (Cai *et al.* 2014; Santhanam *et al.* 2015).

## ncRNA abundances correlate with mRNAs involved in early and late development

Since lncRNAs are abundant throughout development, we propose that they might contribute to cellular and morphological phenotypes. We searched for lncRNAs and mRNAs with strongly correlated transcription profiles (Childs *et al.* 2011; Okamura *et al.* 2015) (Fig 3). We found two groups of correlated transcripts: those abundant, or “on,” early and “off” late; and those off early and on late. Considering decades of characterization of the cell biology of development over time (Kessin 2001), we propose that the early-on lncRNAs are involved in growth (measured at 0h) and in the starvation response at the onset of development, whereas the late-on lncRNAs may contribute to culmination, sporulation and fruiting body maturation. Noncoding transcripts are also present at mid-development time points, (e.g. between hours 8 and 14), leaving open the possibility of roles in multicellular integration and differentiation (Williams 2006; Rosengarten *et al.* 2013).

We wished to test directly whether lncRNAs played a functional role in development. From a collection of barcoded insertion mutants (Robery *et al.* 2013), we identified 15 strains with lesions putatively mapped in or adjacent to asRNAs and lncRNAs (File S8). We grew the strains in association with bacteria on nutrient agar plates and observed growth and development of individual plaques. None of the strains showed overt growth impairment or defects in development on cleared agar. A subset of six strains also developed normally on nitrocellulose filters (data not shown). Our failure to recover lncRNA mutants with obvious phenotypes is likely a reflection of the small sample size of known mutants available for testing. Future genetics studies should be mindful that mutations between coding regions may in fact hit functional genetic elements, and should not be discarded as off-target until the expression of that noncoding region is assessed in a wild-type background.

## Strong temporal and strand signal of an antisense transcript from the mitochondrial genome

*D. discoideum* transcribes its mitochondrial genome (mtDNA) from a single initiation site, with all genes on the same strand (Le *et al.* 2009) (Fig 4a). The resulting polycistronic RNA is processed into 8 smaller multi-genic units, which are further processed into individual gene transcripts (mtRNAs) (Barth *et al.* 2001; Le *et al.* 2009). The transcripts are not poly-adenylated, and therefore have not been included in previously published RNA-seq studies that relied on poly-A library enrichment methods. Overall, mtRNA was highly abundant at the onset of starvation and early development, and declined over the developmental time course (Fig 4b). Even the small and large ribosomal subunit genes, which were targeted for depletion during library preparation, retained high abundance values. This result suggests a limitation of the success of the enzymatic depletion method (Adiconis *et al.* 2013), and speaks to the sheer abundance of mtRNAs overall.

We identified two putative asRNAs mapped to the mtDNA (Fig 4a, green boxes). These overlap with the gene models for (*rnaS/DDB_G0305150*, *trnM, trnL,* and *trnR*) and (*trnP, atp9, trnM,* and *nad9*). The median abundance of the asRNA models was 16-fold lower than that of the top-strand genes. The antisense transcript opposite the *rnaS* locus sharply peaked in abundance at 14 hours (Fig 4b, red box). This peak did not appear to be correlated with the expression of any other mtRNA. The locus including this asRNA model was also notable for a strand specificity of 0.79, well below the genome median, providing additional confidence that this asRNA is independently transcribed. This time point coincides with a major mtDNA replication event during multicellular differentiation (Shaulsky and Loomis 1995). asRNA has been shown to regulate the replication of plasmids in various prokaryotic systems (Brantl 2002, 2015). Whether or not the uptick in asRNA abundance is related to mtDNA replication in *Dictyostelium*, rather than a simple coincidence of small sample size, remains to be tested.

An additional consequence of profiling *Dictyostelium’s* mtRNA is the identification of an annotation issue regarding the small ribosomal subunit (*rnsA*). The annotation of the AX4 mtDNA on dictyBase (version 2013) (Basu *et al.* 2013) does not include the *rnsA* gene, but rather the model *DDB_G0305150* for a gene of unknown function similar to a bacterial protein. Meanwhile, the NCBI mitochondrial genome record (GenBank: AB000109.1), derived from strain AX3, does include the *rnsA* gene annotation overlapping this position. The mtDNA sequences from these two databases display 100% identity (BLAST results not shown), but slight differences in annotation. Our data from AX4 support continuous transcription across the *rnsA* region. Considering the sequence similarity of this locus to small ribosomal subunit genes of other taxa, we propose the mtDNA annotations should be reconciled to include the *rnsA* gene in all cases.

## Abundance and modifications of tRNA transcripts

Although earlier studies cataloged several types of small ncRNAs (Aspegren *et al.* 2004; Avesson *et al.* 2011), the developmental expression of tRNAs in *D. discoideum* has not been described in detail. The present study thus far has focused on long intergenic and antisense transcripts, and the sample preparation method (Ovation, Nugen) was well suited to isolating these molecules. While we detected plenty of reads likely from tRNAs in this library as well, we were not confident in the quantification of the tRNAs because the Ovation method was not optimized to retain molecules smaller than 100 bp. In order to examine tRNAs, typically 70 – 80 bp, we processed a subset of samples—six time points from biological replicate 1—using the riboZero (Epicentre, Madison) rRNA depletion method.

A majority of tRNA gene families is represented by more than a single copy in the *D. discoideum* genome. A total of 418 tRNA genes have been modeled, 403 of which reside on the nuclear genome (<http://dictybase.org/>, version 2013) (Eichinger *et al.* 2005; Fey *et al.* 2009; Basu *et al.* 2013). These correspond to tRNA families with specificity for 41 codons. The prevalence of duplicated loci suggests gene copy number may influence tRNA abundance and availability. With 22 loci each, “tRNA-Lys-UUU” and “tRNA-Asp-GUC” are the most repeated of the tRNA genes. However, the codons they decode, (AAA) and (GAC) respectively, are not the most abundant. Due to the multi-copy state of most tRNA genes, we created a new reference genome with each tRNA allele represented as a contig. We quantified their transcript abundance as the cumulative abundance of all tRNA genes belonging to a tRNA family. Unlike mRNAs and other non-coding RNAs, tRNA abundance didn’t change dramatically during development. So for all further analyses, we aggregated data from all the developmental time points.

We asked whether the abundance of tRNAs correlated with the frequency of the matching codon in the open reading frames throughout the genome (Fig 5a). We found that tRNA abundance and codon frequency were weakly positively correlated (Spearman’s correlation = 0.35), similar to observations in other organisms (reviewed in (Novoa and Pouplana 2012)). Surprisingly, we found that 20 codons had no cognate tRNA partner encoded in the genome.

We wondered how the 20 codons with no tRNA interacting partners might be translated. Based on anticodon similarities, we suspect that 12 of these 20 could be translated through canonical wobble interactions with tRNA isoacceptors (Crick 1966) (data not shown). However, the remaining eight codons with missing tRNA partners are unlikely to be translated merely through classical wobble interactions (Fig 5a). These eight unrepresented tRNA-codon pairs included the least frequent codon, CGG (Arginine), but also more highly ranked codons such as ACC (Threonine, 28^th^ most frequent) and AUC (Isoleucine, 34^th^ most frequent). Transcripts of genes that contain these unmatched codons could suffer from stalled translation, possibly leading to endonucleolytic cleavage and “no-go” mRNA decay (Doma and Parker 2006), or potentially truncated protein expression, which could be detrimental to the cells. One simple explanation might be that transcripts containing these codons are not transcribed, or if transcribed, not translated. We examined 11830 coding sequences obtained from dictyBase (<http://dictybase.org>, version 2013), recently confirmed to be polyadenylated (Cai *et al.* 2014). More than 98% contained at least one of these 8 codons, arguing against the hypothesis that these codons are un-expressed.

As an alternative, we hypothesized that cells resolve the issue of missing codon-tRNA partners through specific post-transcriptional editing of the tRNA to modify the anticodon specificity (Gerber and Keller 1999; Rubio *et al.* 2007; Jackman and Alfonzo 2013). tRNAs are subject to myriad biochemical modifications, including deamination (Jackman and Alfonzo 2013). One of these modifications is deamination of the 5’-adenosine in the anticodon, converting adenosine to inosine, a guanosine analog capable of pairing with (A), (C) or (U) (Gerber and Keller 1999). These modifications expand the decoding capacity of tRNAs to recognize rare codons or compensate for absent isoacceptors. We searched the tRNA transcriptome for evidence of single nucleotide polymorphisms (SNPs) that might modify anticodon specificity. We identified variations within the anticodon that could compensate for four of the eight missing tRNAs (Fig 5b). For example, in the case of the missing tRNA specific for Leucine (CUC), we observed variations in the anti-codon in transcripts of two tRNA-Leu-AAG alleles (Fig 5c, d). In both, the anticodon (AAG) was modified to (GAG). In one case we detected evidence for polymorphism only at the first base of the anticodon (Fig 5c), whereas in the second we additionally identified a SNP at position 28 (Fig 5d). This position, outside of the anticodon, was predicted to remain unpaired in putative secondary structures of the tRNA (S6 Fig) (Lowe and Eddy 1997; Schattner *et al.* 2005). All of the anticodon SNPs involved an A 🡪 G transition, and since inosine is read as guanosine by most sequencing technologies, we interpret this to be evidence of A 🡪 I deamination. Specialized protocols to test if these editing events in fact result in inosine coupled with higher-depth of coverage may be necessary to further validate our findings (Cattenoz *et al.* 2013).

Overall, 27 other tRNAs were found with SNPs somewhere in the transcript (File S9) and many tRNA genes contained SNPs at more than one position. In contrast to the anticodon variants, most other SNPs result in changes of either an (A) or (G) to a (T), suggesting the activity of other post-transcriptional modification mechanisms, or tolerance of some amount of uncorrected transcriptional error in specific positions on tRNAs. We can only speculate at the effect of these polymorphisms, perhaps involved in folding efficiency, ribosome interactions, or amino acid loading. Deeper sampling would be necessary to assess whether post-transcriptional tRNA variation is developmentally regulated. Further functional studies might examine how these changes influence protein expression.

# Conclusion

## Dynamic developmental expression of lncRNAs

We conclude that *D. discoideum* expresses intergenic long noncoding RNAs throughout development, and that the abundance of these molecules changes over developmental time with a trajectory similar to that of mRNAs. The prevalence of these transcripts and the similarity in expression profiles to that of the protein coding transcriptome suggest that lncRNAs are relevant to the progression of molecular and cellular phenotypes from single-celled amoebae to multicellular reproductive fruiting bodies. Hypothesized cis- or trans- interactions between lncRNAs and mRNAs add a layer of complexity to the transcriptional regulatory landscape. Further, post-transcriptional tRNA modifications may play an important part in ensuring timely translation of expressed genes. The catalog of transcripts described in this study sets the stage for future functional studies to decode the functions of ncRNAs in *Dictyostelium*.

## Data resources for future studies

In order to facilitate future analysis of transcriptional links between mRNAs and lncRNAs, we developed a new visualization module on the *Dictyostelium* gene expression atlas dictyExpress ([www.dictyExpress.org](http://www.dictyExpress.org)) (Rosengarten *et al.* Submitted; Rot *et al.* 2009; Stajdohar *et al.* 2015). When a user selects an mRNA or ncRNA and then searches for similar temporal expression profiles, ncRNAs are included in the results. When one or more ncRNAs are then selected, a parallel time course is plotted so that the transcription profiles may be compared, with the abundance (y-axis) appropriately scaled (S7 Fig). This tool will allow the *Dictyostelium* research community to consider ncRNAs when generating hypotheses to test regarding their genes of interest.

# Acknowledgments

The authors would like to thank Adam Kuspa and Mariko Katoh-Kurasawa for helpful discussions and commentary; Frank Tansley and Luke Sherlin from NuGen for advice, guidance and material support; Christopher Dinh for help with identifying REMI mutant strains and Pamela Beltran for technical assistance; Shan Song and Rui Chen for access to materials and sequencing advice; and Lisa White and the Baylor College of Medicine RNA-sequencing core for generously including us in the GARP Ribo-Zero pilot study. We are especially grateful to the team at Genialis for providing excellent visual analytics software to accommodate the requirements of this study. RDR was supported in part by the Keck Center of the Gulf Coast Consortia, Training Program in Biomedical Informatics, National Library of Medicine (T15LM007093-21, PI Tony Gorry, Rice University). Research reported in this publication was supported by the Eunice Kennedy Shriver National Institute Of Child Health & Human Development of the National Institutes of Health under Award Number P01HD039691. The content is solely the responsibility of the authors and does not necessarily represent the official views of the National Institutes of Health.

# Declarations

The authors declare no competing interests. No humans or animals, nor data derived from those sources, were used in this study. Therefore statement of informed consent or IRB approval is not applicable.

# References

Adiconis, X., D. Borges-Rivera, R. Satija, D. S. DeLuca, M. A. Busby *et al.*, 2013 Comparative analysis of RNA sequencing methods for degraded or low-input samples. Nat. Methods 10: 623–629.

Andrews, S., 2010 *FastQC: A quality control tool for high throughput sequence data.*

Aspegren, A., A. Hinas, P. Larsson, A. Larsson, and F. Söderbom, 2004 Novel non-coding RNAs in Dictyostelium discoideum and their expression during development. Nucleic Acids Res. 32: 4646–4656.

Avesson, L., J. Reimegård, E. G. H. Wagner, and F. Söderbom, 2012 MicroRNAs in Amoebozoa: deep sequencing of the small RNA population in the social amoeba Dictyostelium discoideum reveals developmentally regulated microRNAs. RNA N. Y. N 18: 1771–1782.

Avesson, L., H. T. Schumacher, P. Fechter, P. Romby, U. Hellman *et al.*, 2011 Abundant class of non-coding RNA regulates development in the social amoeba Dictyostelium discoideum. RNA Biol. 8: 1094–1104.

Barth, C., U. Greferath, M. Kotsifas, Y. Tanaka, S. Alexander *et al.*, 2001 Transcript mapping and processing of mitochondrial RNA in Dictyostelium discoideum. Curr. Genet. 39: 355–364.

Basu, S., P. Fey, Y. Pandit, R. Dodson, W. A. Kibbe *et al.*, 2013 dictyBase 2013: integrating multiple Dictyostelid species. Nucleic Acids Res. 41: D676–D683.

Bernstein, P., S. W. Peltz, and J. Ross, 1989 The poly(A)-poly(A)-binding protein complex is a major determinant of mRNA stability in vitro. Mol. Cell. Biol. 9: 659–670.

Brantl, S., 2015 Antisense-RNA mediated control of plasmid replication - pIP501 revisited. Plasmid 78: 4–16.

Brantl, S., 2002 Antisense RNAs in plasmids: control of replication and maintenance. Plasmid 48: 165–173.

Broadbent, K. M., J. C. Broadbent, U. Ribacke, D. Wirth, J. L. Rinn *et al.*, 2015 Strand-specific RNA sequencing in Plasmodium falciparum malaria identifies developmentally regulated long non-coding RNA and circular RNA. BMC Genomics 16: 454.

Cai, H., M. Katoh-Kurasawa, T. Muramoto, B. Santhanam, Y. Long *et al.*, 2014 Nucleocytoplasmic shuttling of a GATA transcription factor functions as a development timer. Science 343: 1249531.

Cattenoz, P. B., R. J. Taft, E. Westhof, and J. S. Mattick, 2013 Transcriptome-wide identification of A > I RNA editing sites by inosine specific cleavage. RNA 19: 257–270.

Childs, K. L., R. M. Davidson, and C. R. Buell, 2011 Gene coexpression network analysis as a source of functional annotation for rice genes. PloS One 6: e22196.

Crick, F. H., 1966 Codon--anticodon pairing: the wobble hypothesis. J. Mol. Biol. 19: 548–555.

Demšar, J., T. Curk, A. Erjavec, Č. Gorup, T. Hočevar *et al.*, 2013 Orange: Data Mining Toolbox in Python. J. Mach. Learn. Res. 14: 2349–2353.

Derrien, T., R. Johnson, G. Bussotti, A. Tanzer, S. Djebali *et al.*, 2012 The GENCODE v7 catalog of human long noncoding RNAs: Analysis of their gene structure, evolution, and expression. Genome Res. 22: 1775–1789.

Doma, M. K., and R. Parker, 2006 Endonucleolytic cleavage of eukaryotic mRNAs with stalls in translation elongation. Nature 440: 561–564.

Eichinger, L., J. A. Pachebat, G. Glöckner, M.-A. Rajandream, R. Sucgang *et al.*, 2005 The genome of the social amoeba Dictyostelium discoideum. Nature 435: 43–57.

Fey, P., P. Gaudet, T. Curk, B. Zupan, E. M. Just *et al.*, 2009 dictyBase--a Dictyostelium bioinformatics resource update. Nucleic Acids Res. 37: D515–519.

Gerber, A. P., and W. Keller, 1999 An Adenosine Deaminase that Generates Inosine at the Wobble Position of tRNAs. Science 286: 1146–1149.

Guil, S., and M. Esteller, 2012 Cis-acting noncoding RNAs: friends and foes. Nat. Struct. Mol. Biol. 19: 1068–1075.

Haas, B. J., A. Papanicolaou, M. Yassour, M. Grabherr, P. D. Blood *et al.*, 2013 De novo transcript sequence reconstruction from RNA-seq using the Trinity platform for reference generation and analysis. Nat. Protoc. 8: 1494–1512.

Hildebrandt, M., and W. Nellen, 1992 Differential antisense transcription from the Dictyostelium EB4 gene locus: implications on antisense-mediated regulation of mRNA stability. Cell 69: 197–204.

Hinas, A., P. Larsson, L. Avesson, L. A. Kirsebom, A. Virtanen *et al.*, 2006 Identification of the major spliceosomal RNAs in Dictyostelium discoideum reveals developmentally regulated U2 variants and polyadenylated snRNAs. Eukaryot. Cell 5: 924–934.

Hinas, A., and F. Söderbom, 2007 Treasure hunt in an amoeba: non-coding RNAs in Dictyostelium discoideum. Curr. Genet. 51: 141–159.

Iranfar, N., D. Fuller, and W. F. Loomis, 2003 Genome-wide expression analyses of gene regulation during early development of Dictyostelium discoideum. Eukaryot. Cell 2: 664–670.

Jackman, J. E., and J. D. Alfonzo, 2013 Transfer RNA modifications: nature’s combinatorial chemistry playground. Wiley Interdiscip. Rev. RNA 4: 35–48.

Kessin, R. H., 2001 *Dictyostelium: evolution, cell biology, and development of multicellularity*. Cambridge University Press.

Koboldt, D. C., Q. Zhang, D. E. Larson, D. Shen, M. D. McLellan *et al.*, 2012 VarScan 2: Somatic mutation and copy number alteration discovery in cancer by exome sequencing. Genome Res. 22: 568–576.

Kornienko, A. E., P. M. Guenzl, D. P. Barlow, and F. M. Pauler, 2013 Gene regulation by the act of long non-coding RNA transcription. BMC Biol. 11: 59.

Krzywinski, M., J. Schein, I. Birol, J. Connors, R. Gascoyne *et al.*, 2009 Circos: an information aesthetic for comparative genomics. Genome Res. 19: 1639–1645.

Langmead, B., and S. L. Salzberg, 2012 Fast gapped-read alignment with Bowtie 2. Nat. Methods 9: 357–359.

Larsson, P., A. Hinas, D. H. Ardell, L. A. Kirsebom, A. Virtanen *et al.*, 2008 De novo search for non-coding RNA genes in the AT-rich genome of Dictyostelium discoideum: Performance of Markov-dependent genome feature scoring. Genome Res. 18: 888–899.

Le, P., P. R. Fisher, and C. Barth, 2009 Transcription of the Dictyostelium discoideum mitochondrial genome occurs from a single initiation site. RNA N. Y. N 15: 2321–2330.

Li, H., B. Handsaker, A. Wysoker, T. Fennell, J. Ruan *et al.*, 2009 The Sequence Alignment/Map format and SAMtools. Bioinforma. Oxf. Engl. 25: 2078–2079.

Liu, S. J., T. J. Nowakowski, A. A. Pollen, J. H. Lui, M. A. Horlbeck *et al.*, 2016 Single-cell analysis of long non-coding RNAs in the developing human neocortex. Genome Biol. 17: 67.

Loomis, W. F., and G. Shaulsky, 2011 Developmental changes in transcriptional profiles. Dev. Growth Differ. 53: 567–575.

Lowe, T. M., and S. R. Eddy, 1997 tRNAscan-SE: a program for improved detection of transfer RNA genes in genomic sequence. Nucleic Acids Res. 25: 955–964.

Miranda, E. R., G. Rot, M. Toplak, B. Santhanam, T. Curk *et al.*, 2013 Transcriptional profiling of dictyostelium with RNA sequencing, in *Dictyostelium discoideum Protocols*, Methods in Molecular Biology, Springer.

Miranda, E. R., O. Zhuchenko, M. Toplak, B. Santhanam, B. Zupan *et al.*, 2013 ABC transporters in Dictyostelium discoideum development. PloS One 8: e70040.

Novoa, E. M., and L. R. de Pouplana, 2012 Speeding with control: codon usage, tRNAs, and ribosomes. Trends Genet. 28: 574–581.

Okamura, Y., T. Obayashi, and K. Kinoshita, 2015 Comparison of Gene Coexpression Profiles and Construction of Conserved Gene Networks to Find Functional Modules. PloS One 10: e0132039.

Parikh, A., E. R. Miranda, M. Katoh-Kurasawa, D. Fuller, G. Rot *et al.*, 2010 Conserved developmental transcriptomes in evolutionarily divergent species. Genome Biol. 11: R35.

Pelechano, V., and L. M. Steinmetz, 2013 Gene regulation by antisense transcription. Nat. Rev. Genet. 14: 880–893.

Qu, Z., and D. L. Adelson, 2012 Identification and comparative analysis of ncRNAs in human, mouse and zebrafish indicate a conserved role in regulation of genes expressed in brain. PloS One 7: e52275.

Quinn, J. J., and H. Y. Chang, 2016 Unique features of long non-coding RNA biogenesis and function. Nat. Rev. Genet. 17: 47–62.

Raper, K., 1940 Pseudoplasmodium formation and organization in Dictyostelium discoideum. J. Elisha Mitchell Sci. Soc. 56: 241–282.

Rinn, J. L., and H. Y. Chang, 2012 Genome regulation by long noncoding RNAs. Annu. Rev. Biochem. 81: 145–66.

Robery, S., R. Tyson, C. Dinh, A. Kuspa, A. A. Noegel *et al.*, 2013 A novel human receptor involved in bitter tastant detection identified using Dictyostelium discoideum. J Cell Sci 126: 5465–5476.

Robinson, J. T., H. Thorvaldsdóttir, W. Winckler, M. Guttman, E. S. Lander *et al.*, 2011 Integrative genomics viewer. Nat. Biotechnol. 29: 24–26.

Rosengarten, R. D., P. R. Beltran, and G. Shaulsky, 2015b A deep coverage Dictyostelium discoideum genomic DNA library replicates stably in Escherichia coli. Genomics.

Rosengarten, R. D., J. Kokošar, L. Jeran, G. Shaulsky, B. Zupan *et al.*, Submitted dictyExpress: a Web-Based Platform for Sequence Data Management and Analytics in Dictyostelium and Beyond.

Rosengarten, R. D., B. Santhanam, D. Fuller, M. Katoh-Kurasawa, W. F. Loomis *et al.*, 2015a Leaps and lulls in the developmental transcriptome of Dictyostelium discoideum. BMC Genomics 16: 294.

Rosengarten, R. D., B. Santhanam, and M. Katoh-Kurasawa, 2013 Transcriptional Regulators: Dynamic Drivers of Multicellular Formation, Cell Differentiation and Development, pp. 89–108 in *Dictyostelids*, Springer.

Rot, G., A. Parikh, T. Curk, A. Kuspa, G. Shaulsky *et al.*, 2009 dictyExpress: a Dictyostelium discoideum gene expression database with an explorative data analysis web-based interface. BMC Bioinformatics 10: 265.

Rubio, M. A. T., I. Pastar, K. W. Gaston, F. L. Ragone, C. J. Janzen *et al.*, 2007 An adenosine-to-inosine tRNA-editing enzyme that can perform C-to-U deamination of DNA. Proc. Natl. Acad. Sci. 104: 7821–7826.

Sachs, A., 1990 The role of poly(A) in the translation and stability of mRNA. Curr. Opin. Cell Biol. 2: 1092–1098.

Santhanam, B., H. Cai, P. N. Devreotes, G. Shaulsky, and M. Katoh-Kurasawa, 2015 The GATA transcription factor GtaC regulates early developmental gene expression dynamics in Dictyostelium. Nat. Commun. 6: 7551.

Schattner, P., A. N. Brooks, and T. M. Lowe, 2005 The tRNAscan-SE, snoscan and snoGPS web servers for the detection of tRNAs and snoRNAs. Nucleic Acids Res. 33: W686–689.

Shaulsky, G., and W. F. Loomis, 1995 Mitochondrial DNA replication but no nuclear DNA replication during development of Dictyostelium. Proc. Natl. Acad. Sci. U. S. A. 92: 5660–5663.

Stajdohar, M., L. Jeran, J. Kokošar, D. Blenkus, T. Janez *et al.*, 2015 *dictyExpress: visual analytics of NGS gene expression in Dictyostelium*.

Sucgang, R., G. Chen, W. Liu, R. Lindsay, J. Lu *et al.*, 2003 Sequence and structure of the extrachromosomal palindrome encoding the ribosomal RNA genes in Dictyostelium. Nucleic Acids Res. 31: 2361–2368.

Suzuki, R., and H. Shimodaira, 2006 Pvclust: an R package for assessing the uncertainty in hierarchical clustering. Bioinformatics 22: 1540–1542.

Takeishi, K., and S. Kaneda, 1981 Isolation and characterization of small nuclear RNAs from Dictyostelium discoideum. J. Biochem. (Tokyo) 90: 299–308.

Takeishi, K., and S. Kaneda, 1979 Low molecular weight nuclear RNA species in Dictyostelium discoideum. Nucleic Acids Symp. Ser. s125–127.

Thorvaldsdóttir, H., J. T. Robinson, and J. P. Mesirov, 2013 Integrative Genomics Viewer (IGV): high-performance genomics data visualization and exploration. Brief. Bioinform. 14: 178–192.

Trapnell, C., L. Pachter, and S. L. Salzberg, 2009 TopHat: discovering splice junctions with RNA-Seq. Bioinforma. Oxf. Engl. 25: 1105–1111.

Trapnell, C., A. Roberts, L. Goff, G. Pertea, D. Kim *et al.*, 2012 Differential gene and transcript expression analysis of RNA-seq experiments with TopHat and Cufflinks. Nat. Protoc. 7: 562–578.

Van Driessche, N., J. Demsar, E. O. Booth, P. Hill, P. Juvan *et al.*, 2005 Epistasis analysis with global transcriptional phenotypes. Nat. Genet. 37: 471–477.

Van Driessche, N., C. Shaw, M. Katoh, T. Morio, R. Sucgang *et al.*, 2002 A transcriptional profile of multicellular development in Dictyostelium discoideum. Development 129: 1543–1552.

Wang, Z., N. Day, P. Trifillis, and M. Kiledjian, 1999 An mRNA Stability Complex Functions with Poly(A)-Binding Protein To Stabilize mRNA In Vitro. Mol. Cell. Biol. 19: 4552–4560.

Williams, J. G., 2010 Dictyostelium finds new roles to model. Genetics 185: 717–726.

Williams, J. G., 2006 Transcriptional regulation of Dictyostelium pattern formation. EMBO Rep. 7: 694–698.

# Figure Legends

**Fig 1. Long noncoding RNAs are distributed throughout the genome and characteristically distinct from annotated mRNAs.** (a) Transcript density is shown on a Circos plot. The outer-most ring, shaded solid gray, represents the scale of each chromosome, in megabase pairs (MB). The six chromosomes and the ribosomal palindrome are labeled inside the plot (1 – 6, R). The black bars/white spaces on chromosomes 2 and R mark large duplications for which reads are mapped only to one region. Transcript density was calculated as the percentage of base pairs per 10 kilobase pair window that mapped to messenger (m)RNA (grey, outer plot) and intergenic long non-coding (lnc)RNA (red, inner plot). Each plot is scaled from 0 to 1, with top-strand transcripts above the zero-axis, and bottom-strand transcripts below this axis. The distributions of (b) maximum transcript abundance, (c) transcript length and (d) GC content are shown on the y-axis of the box and whisker plots, with RNA type on the x-axis. In all cases, the box height represents the 1^st^ to 3^rd^ quartiles and the horizontal line, the median value. Whisker bars mark 1.5-fold the 1^st^/3^rd^ quartile range, with outliers displayed as circles. (b) Maximum transcript abundance was determined for each transcript model across all developmental time points, and is plotted on a log scale. (c) Transcript length was also log scaled. (d) For the comparison of GC content, in addition to the three RNA classes, we randomly sampled 1,000 intergenic regions per chromosome that did not overlap with any transcript model. All four distributions were significantly different. For plots (b – d), p-values were determined by Mann-Whitney U-test.

**Fig 2. Noncoding RNAs are developmentally regulated as well as mRNAs.** (a ,b) Heatmaps reveal temporal changes in RNA abundance throughout development. The heatmap rows each represent a gene/transcript model, clustered based on their transcript abundance, and columns correspond to sample time points (2-hour intervals), increasing from left to right. RNA abundance values are represented as row-wise Z-scores and are color coded as indicated in the scale above each heatmap. (c,d) Multidimensional scaling shows the distances between transcriptomes at each time point for each class of RNA. Dimension 1 is on the x-axis and dimension 2 on the y-axis, and distances between points (arbitrary units, not shown) on the 2-D plane are inversely proportional to the similarity (Spearman’s correlation) of the transcriptomes. Black circles represent sample averages with the time point labeled, while individual biological replicates 1 and 2 are shown as open and grey circles, respectively, connected by whiskers. Only replicate 1 for time points 16 and 22 passed our quality control, and thus these are shown as labeled open circles. (e,f) Plots of Dimension 1 values (arbitrary units, y-axis) versus time (hours, x-axis) for mRNA (e) and lncRNA (f). The dotted diagonal represents a linear best-fit curve, with coefficients of determination (r^2^) displayed on each plot.

**Fig 3. Subsets of lncRNAs are strongly correlated with mRNAs.** The purple/green heatmap represents a matrix that relates the transcription profiles of 51 abundant lncRNAs (columns: x-axis dendrogram, bottom heatmap) with 858 developmentally regulated mRNAs (rows: y-axis dendrogram, side heatmap). Spearman’s correlation coefficients (false discovery rate < 0.01) are shown in green (≥0.85, positive correlation) and purple (≤-0.85, negative correlation) as indicated by the green/white/purple scale bar. Dendrograms display hierarchical clustering of transcripts based on their abundance during development, which is shown in the blue/yellow heatmaps. The color range is scaled to the row-wise Z-distribution as indicated by the blue/yellow scale bar. In these heatmaps, one axis represents developmental time points, from 0 to 24 hours in 2-hour increments as indicated, and the other axis represents individual transcripts.

**Fig 4. The mitochondrial transcriptome.** (a) All open reading frames (gray boxes), including genes encoding ribosomal proteins and ribosomal RNA (rRNA) subunits (dark gray boxes), and tRNAs (blue boxes) are transcribed in the same, clockwise direction. Genes that overlap other models are offset. The red tick (around 4 o’clock) indicates the single transcription start site. Distances are marked in kilobases (kb) inside the circle. Antisense RNA transcripts are shown as green boxes. Median read coverage, calculated for 100bp sliding windows is shown as a histogram (purple bars) inside of the gene track. The values were log_2_ transformed for scale, and the plot ranges from 0 to 15. Although rRNAs (**) were among the most abundant transcripts, these molecules were targeted for depletion, so this read density likely does not reflect the true abundances. (b) A heatmap of mtRNA expression reveals declining abundance of most transcripts, and a spike in asRNA at 14 hours, during development. Rows each represent a gene/transcript model, sorted by similarity, and columns correspond to sample time points (2-hour intervals), increasing from 0 hours on the left to 24 hours on the right. RNA abundance values are represented as row-wise Z-scores and are color coded as indicated in the scale above the heatmap. Antisense RNAs are marked with a red box.

**Fig 5. Post-transcriptional modifications of tRNAs may compensate for some codon-specificity missing from the genome.** (a) We plotted the rank order (1 is the lowest) of tRNA transcript abundance (circles, y-axis) versus the rank order of the codon frequency in the *D. discoideum* exome (x-axis). The stop codons were removed, thus the axes scale to 61 rather than 64. The dotted red line represents the y=x line. Twenty codons do not have matching tRNAs in the genome, thus the developmental abundance of the cognate tRNA was 0 (rank = 1; parallel to X-axis), whereas the rest were ranked 21 to 61. Eight of the unmatched tRNA-codon pairs, which we hypothesized undergo post-transcriptional editing, are shown as larger colored circles identified in the legend. They are annotated with the amino acid specificity, followed by (CODON/ anticodon, codon frequency %). (b) Statistical support for the detection of anti-codon modifications that mimic four of the eight unmatched tRNAs is shown as –log_10_(p-value) on the y-axis. The horizontal dotted line corresponds to a p-value = 0.05. The y-axis is discontinuous between values 25 and 80. The cognate tRNA for the codon CUU (tRNA-Leu-AAG) has two alleles encoded in the genome that undergo editing events in their anti-codon region (red circles) (c) and (d). Both had a significant modification detected at base 36, the first position of the anticodon. Allele 2 (d) had a second modification at base 28. Modifications are marked by vertical black bars, the height of which indicates the statistical support (y-axis). The positions of the modifications are indicated on the x-axis. The horizontal dotted line corresponds to a p-value of 0.05.

# Supporting Files

**Supplemental Figures.** A PDF file containing Supplemental Figures 1 – 7 and legends.

**File S1.** Custom designed InDA-C oligos for rRNA depletion.

**File S2.** Cuffmerge output of the raw transcriptome assembly.

**File S3.** Expressions of previous DDB_G gene models and curated CUFF models.

**File S4.** Curated list of asRNA and lncRNAs transcripts and their newly assigned corresponding DDB_G gene models.

**File S5.** mRNA expression values used to compute Spearman’s correlations between experimental methods.

**File S6.** Spearman’s correlations and strand orientations between lncRNAs and nearest neighboring genes in either direction.

**File S7.** Spearman’s correlations, strand specificity and intron overlap of asRNA models and sense strand genes.

**File S8.** REMI mutants tested for developmental phenotypes due to lncRNA disruptions.

**File S9.** Varscan output including all detected tRNA polymorphisms.

1. Current address: Genialis Inc., Houston, TX 77005, USA [↑](#footnote-ref-1)
